# Combined emapalumab and ruxolitinib in patients with haemophagocytic Lymphohistiocytosis

**DOI:** 10.1038/s41408-024-01056-0

**Published:** 2024-04-24

**Authors:** Yue Song, Fei Zhou, Feng Du, Ziyan Wang, Liyun Bai, Yifang Yao, Limin Liu, Xiao Ma, Suning Chen, Depei Wu, Xuefeng He

**Affiliations:** 1 National Clinical Research Center for Hematologic Diseases, Jiangsu Institute of Hematology, The First Affiliated Hospital of Soochow University, Suzhou, 215006 China; 2 Institute of Blood and Marrow Transplantation, Collaborative Innovation Center of Hematology, Soochow University, Suzhou, 215031 China; 3 Department of hematology, Soochow Hopes Hematonosis Hospital, Suzhou, 215128 Jiangsu China

**Keywords:** Haematological diseases, Immunological disorders, Immunotherapy

**TO THE EDITOR:**

Haemophagocytic lymphohistiocytosis (HLH) is a life-threatening systemic inflammatory disorder characterized by excessive activation of cytotoxic T cells and macrophages [1, 2]. Cytokines play a pivotal role in the progression of HLH and are thus mainly targeted in immunotherapy. IFN-γ is considered the most crucial cytokine in HLH pathology. Based on the positive outcomes of a clinical trial using an anti-IFN-γ antibody in patients with primary HLH (pHLH), the IFN-γ neutralizing antibody, emapulumab (Gamifant®), has received FDA approval for treating refractory or recurrent pHLH or those intolerant to conventional HLH therapies [3]. However, the need for the addition of other therapies like dexamethasone implies that inhibiting IFN-γ alone may not be sufficient to control the disease in a significant proportion of patients.

Most of the cytokines elevated in HLH signal through the Janus Kinases (JAK) and Signal Transducers and Activators of Transcription (STAT) pathway [4]. Ruxolitinib, a JAK 1/2 inhibitor, has proven effective in HLH by dampening downstream signaling of numerous cytokines [5, 6]. However, previous clinical studies of ruxolitinib in HLH showed only partial effects, with few cases achieving complete remission. It is evident that neither IFN-γ blockade nor ruxolitinib monotherapy can lead to complete disease control in HLH. In recent studies, the combination of ruxolitinib and anti-IFN-γ antibody in pHLH animal models demonstrated inconsistent results with various dosage [7–9]. Clearly, there is ongoing debate about whether combining cytokine-targeting agents could have an additive effect in mitigating inflammation in HLH. Considering that the current studies on the combination of ruxolitinib and IFN-γ blockade are all animal experiments, we aim to report the first application of these two agents together in actual clinical patients.

A retrospective analysis of 13 patients diagnosed as HLH who received treatment with emapalumab combined with ruxolitinib at our center was conducted [2]. Patients received emapalumab at a dose of 50–100 mg (~1–2 mg/kg), and ruxolitinib of 20–30 mg/m^2^/day orally. In certain cases, corticosteroid, intravenous immunoglobulin (IVIG), and/or continuous renal replacement therapy were administered along with therapy. Additional chemotherapy was allowed following emapalumab and ruxolitinib if the physicians deemed the response inadequate. Clinical and laboratory evaluations related to HLH were performed before and after therapy (on days 3, 7, 14). Therapeutic response was assessed in accordance with established criteria as previously described (details in supplementary appendix) [3]. Response had to be maintained for at least 3 days to be considered in the analysis. The adverse events related to therapy, the number of patients proceeding to HSCT, 2-month survival, overall survival, and cause of death were also analyzed.

All patients were adults (≥18 years old) with a median age of 34 years (range, 18–79 years), comprising 10 males and 3 females. Demographics and clinical characteristics of the patients were presented in Table 1 and Table S1. There were 8 patients who received previous conventional therapy for HLH, including corticosteroids, HLH-94/04 regimen, and intravenous IVIG in the majority of patients, and four of them had already been receiving ruxolitinib before emapalumab’s intervention. Five of eight patients did not respond to the previous treatment, while the other 3 experienced transient ameliorations followed by rapid relapses. The median time of clinical course, from the estimated onset of HLH symptoms to the first emapalumab infusion, was 4.6 weeks (range, 1.7–13.3 weeks). Five patients received two doses of emapalumab and one patient received four doses. Three patients received concomitant low-dose etoposide (50 mg/m^2^). The median duration of treatment received for emapalumab and ruxolitinib together was 1.14 weeks (IQR 1.0–2.8 weeks) (6.7 weeks (IQR 3.6–9.4 weeks) for ruxolitinib and 1.14 weeks (IQR 1.0–2.8 weeks) for emapalumab, separately).

**Table 1:** Demographics and clinical characteristics of the patients.

Characteristics	Number (*N* = 13)/percentage (%)
Age (years)	
Median (range)	34 (18–79)
Gender-no. (%)	
Female	3 (23)
Male	10 (77)
Aetiology-no. (%)	9 (37.5)
Epstein-Barr virus infection	5 (38)
Lymphoma	5 (38)
Autoimmune disease	2 (15)
Unidentified	1 (8)
Onset of HLH symptoms to therapy (weeks)	
Median (range)	4.6 (1.7–13.3)
Regimens towards HLH prior to emapalumab-no. (%)	
Intravenous glucocorticoids	5 (38)
Ruxolitinib	5 (38)
Etoposide	4 (31)
IVIG	2 (15)
Ciclosporin	1 (8)
Status of HLH before therapy-no. (%)	
Initial	5 (38)
Refractory	5 (38)
Relapse	3 (23)

*IVIG* intravenous immunoglobulin.

Overall, 77% (10/13) of patients responded positively to therapy. Among them, 62% (8/13) achieved remission, with four patients (5, 8, 10, and 11) reaching CR (31%) and another four (3, 4, 9, and 13) achieving PR (31%). Two patients (2 and 7) showed improvement (15%) but did not meet the criteria for remission. In the remaining three patients (1, 6, and 12), two experienced resolutions of fever but did not meet the criteria for improvement. 75% (6/8) of previously treated patients (relapse/refractory) and 80% (4/5) of treatment-naive patients exhibited a positive response (Fig. 1A). Two improved patients and one PR patient underwent additional chemotherapy due to physicians’ consideration. Among the other three PR patients, two experienced disease relapses, with one undergoing allogeneic hematopoietic stem cell transplantation (allo-HSCT) after salvage chemotherapy, and the other succumbing to HLH. Among the three patients with no response, one achieved remission after additional chemotherapy, while the other two died despite showing improvement after additional chemotherapy. Among the four CR patients, one underwent allo-HSCT for long-term survival (EBV-HLH), and the other three survived. Treatment and outcome details are presented in Table S2.

**Fig. 1:**
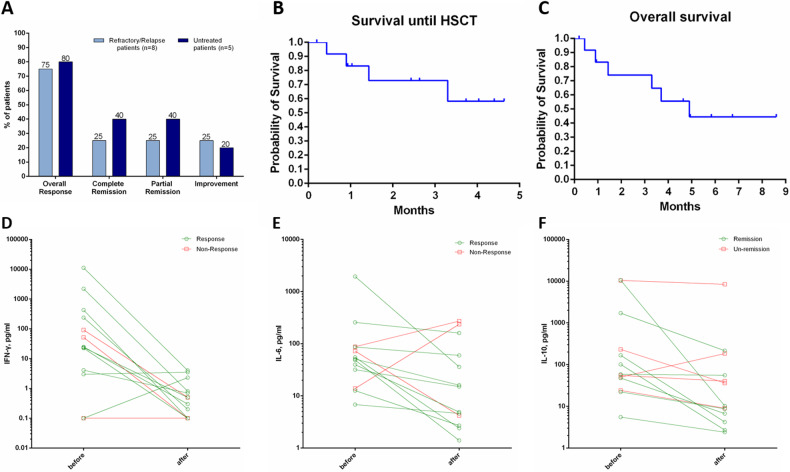
Treatment and outcome details of patients. **A** Overall response of patients. Percentages of patients with a complete response, partial response, or improvement in measures of haemophagocytic lymphohistiocytosis among the previously treated patients and treatment-naive patients. **B** Estimates of the probability of survival until HSCT. **C** Estimates of the probability of overall survival. **D** Levels of Interferon-γ before and after ruxolitinib and emapalumab treatment. **E** Interleukin-6 concentration. **F** Interleukin-10 concentration. The decline in IFN-γ was not associated with disease response. Instead, the decrease in IL-6 and IL-10 correlated with the therapeutic effect: decreased IL-6 levels were associated with a response (*p* = 0.008) and remission (*p* = 0.025), and decreased IL-10 levels were associated with remission (*p* = 0.007) but not a response (*p* = 0.066).

The median follow-up time was 5.8 months (IQR 4.9–6.9 months). In general, 5 patients (38%) proceeded to allo-HSCT, 4 patients died, and 4 patients survived. As for the death cause, three patients died of HLH, and the other one suffered sudden cardiac death, which was unrelated to HLH. The estimated probability of survival from initiation of emapalumab was 72.9% at 2 months. Survival to HSCT is shown in Fig. 1B. Three of five patients underwent allo-HSCT survived until last follow-up, the other two died of HSCT-related complications (infection). The estimated probability of overall survival at 5 months was 44.4%. The overall survival is shown in Fig. 1C.

Almost all patients’ fever resolved within 48 hours (92%, 12/13). Only one patient continued fevering after emapalumab administration (Figure S1). All laboratory parameters of HLH rapidly improved upon the initiation of emapalumab, with evident improvement at week 1 in the majority of patients (Figure). Among the five EBV-HLH patients, four of them also received PD-1 blockade during/after emapalumab, and the intracellular EBV-DNA copies significantly decreased in only one patient (patient 11). Before treatment, IL-6 concentrations were significantly elevated in all patients, and IL-10 concentrations were elevated in 92% (12/13). However, IFN-γ concentrations were variable across patients (elevated in 62%, 8/13). Emapalumab administration resulted in a rapid decrease to normal levels of IFN-γ in all patients. However, the decline in IFN-γ was not associated with disease response (Fig. 1D and S2). Instead, the decrease in IL-6 and IL-10 correlated with the therapeutic effect: decreased IL-6 levels were associated with response (*p* = 0.008) and remission (*p* = 0.025) (Fig. 1E and S3), and decreased IL-10 levels were associated with remission (*p* = 0.007) but not response (*p* = 0.066) (Fig. 1F and S4). No grade 3 or higher drug-related adverse events were observed in any of the patients. Only one patient experienced persistent CMV infection, considered possibly related to emapalumab. The infection resolved after three weeks of standard antiviral treatment. Other patients with pre-existing infections before treatment did not experience exacerbation.

In the last three years, three animal studies investigating the combination of emapalumab and ruxolitinib have been published, but with differences in conclusions [7–9]. This study represents the first clinical application of ruxolitinib and emapalumab combination in HLH, which demonstrated improved response rate compared to ruxolitinib or emapalumab alone. Also, previous clinical trials reported a CR rate of only 21% for mono-emapalumab and 14.7% for mono-ruxolitinib [3, 10]. Complete disease control is crucial for better outcomes of HLH. Although the CR rate was not satisfactory enough in our study, it was still improved (31%). Especially all patients in this study were adults with secondary HLH, predominantly attributed to EBV infection or lymphoma, representing a subset with a challenging prognosis in HLH.

The animal model study conducted by Humblet-Baron et al. [11] proposed a dichotomous pathogenesis in HLH: the hematologic features are dependent on IFN-γ, while the inflammatory aspect is not. A recent study revealed that IFN-γ–knockout PKO mice infected with LCMV still develop full-blown HLH in a neutrophil-dependent manner [12]. Additionally, Brisse et al. indicated that CMV–infected IFN-γ–deficient mice, still developed the HLH-like symptoms [13]. All of these findings cast doubt on the absolute necessity of IFN-γ in the presence of HLH. An altered cytokine milieu dominated by IL-1b, IL-4, IL-6, and GM-CSF may contribute to the immunopathology in IFN-γ-independent-HLH [8]. Ruxolitinib holds the ability to inhibit the signal transduction of these cytokines [5], which is the possible theory for the enhanced efficacy of combination therapy. In our study, patients who responded showed a more significant decrease in IL-6, whereas this phenomenon was not observed in IFN-$\gamma$, supporting the aforementioned theory.

Concerns about the combination therapy, for ruxolitinib, are mainly reported by Jordon et al. [7], who believe that the addition of ruxolitinib worsens myelosuppression. For emapalumab, the main concern lies in the heightened GM-CSF-producing capacity by T cells in the absence of IFN-γ signaling [12, 14]. In our study, the low-dosage agents’ combination proved to have a beneficial effect. The inhibition of JAK2 by ruxolitinib helps control the excessive rise in neutrophil counts triggered by IFN-γ blockade. Moreover, no serious adverse effects were observed. The dosage ents may be a crucial factor. In both animal studies, which concluded the combination appeared to have no advantages, the doses of both agents were rather high. Given that IFN-γ plays important immunoregulatory roles, complete blockade or genetic ablation will exacerbate immune responses following various infections [15]. Therefore, absolute blockade could be detrimental to outcomes, whereas a relatively low dose may be beneficial. Additionally, the relatively low dose of ruxolitinib (4 mg/kg vs 90 mg/kg in mice) avoids adverse effects such as myelosuppression.

In conclusion, the clinical application results demonstrate that the combination of low-dose ruxolitinib and anti-IFN-γ antibodies is a more effective and safe treatment for HLH. This approach shows promise even in cases of EBV- and lymphoma-associated sHLH, which typically suffer a poor prognosis, as well as in relapsed/refractory HLH, offering the possibility of bridging to HSCT. However, the doses and schedules of these agents must be carefully titrated to confer maximum benefit.

### Supplementary information

supplementary appendix

## Data Availability

All relevant raw data, will be freely available to any researcher wishing to use them for non-commercial purposes, without breaching participant confidentiality. For publication-related data, please contact the corresponding author.
