# Supplementary material for: Combined emapalumab and ruxolitinib in patients with haemophagocytic Lymphohistiocytosis
Source: Blood Cancer J. 2024 Apr 24;14(1):70. doi: 10.1038/s41408-024-01056-0 (PMC11043404; doi:10.1038/s41408-024-01056-0)

Table S1. Clinical and laboratory features of HLH before emapalumab and ruxolitinb.

| Case | Gender | Age (years) | Aetiology | Clinical features | ANC (*10^9^/L) | HGB (g/L) | PLT (*10^9^/L) | Ferritin (ng/ml) | sCD25 (pg/ml) | IFN-γ (pg/ml) | EBV-DNA (copies/ml) |
| --- | --- | --- | --- | --- | --- | --- | --- | --- | --- | --- | --- |
| 1 | M | 18 | Lymphoma | Fever, splenomegaly, liver dysfunction | 0.92 | 46 | 2 | 813 | 2701 | 0 | \| <5.0*10^2^ \| \| --- \| |
| 2 | M | 25 | EBV | Fever, splenomegaly, liver dysfunction | 1.87 | 122 | 27 | 53667 | 16239 | 237.6 | <5.0*10^2^ |
| 3 | M | 54 | PTCL | Fever, | 0.48 | 84 | 83 | 3222 | 36321 | 22.5 | 2.37*10^4^ |
| 4 | M | 24 | EBV | Fever, splenomegaly, liver dysfunction | 0.53 | 125 | 33 | 5379 | 28836 | 4.1 | <5.0*10^2^ |
| 5 | F | 63 | Sjogren Syndrome | Fever, splenomegaly | 8.72 | 72 | 184 | 8257 | 2588 | 3 | 1.67*10^3^ |
| 6 | M | 46 | EBV | Fever, splenomegaly | 1.51 | 88 | 72 | 4205 | 21713 | 91.3 | <5.0*10^2^ |
| 7 | M | 34 | Lymphoma | Fever, splenomegaly | 1.76 | 74 | 104 | 2728 | 6520 | 23.5 | <5.0*10^2^ |
| 8 | F | 38 | Lymphoma | Fever, liver dysfunction | 0.84 | 88 | 24 | 191588 | 31817 | 2206.1 | <5.0*10^2^ |
| 9 | M | 33 | EBV | Fever, splenomegaly, liver dysfunction | 0.58 | 74 | 98 | 27508 | 8986 | 24.7 | <5.0*10^2^ |
| 10 | F | 35 | Unkonwn | Fever, splenomegaly, liver dysfunction | 0.06 | 67 | 31 | 30050 | 26081 | 426.5 | <5.0*10^2^ |
| 11 | M | 29 | EBV | Fever, splenomegaly | 0.11 | 82 | 27 | 501367 | 13300 | 11101 | 8.77*10^6^ |
| 12 | M | 33 | NK/T cell lympphoma | Fever, splenomegaly, liver dysfunction | 0 | 66 | 14 | 7815 | 6289 | 51.4 | <5.0*10^2^ |
| 13 | M | 79 | Adult-onset Still’s disease | Fever, liver dysfunction | 0.55 | 63 | 28 | 169575 | 4856 | 0 | <5.0*10^2^ |

F, female; M, male; PTCL, peripheral T-cell lymphoma; ANC, absolute neutrophil count; HGB, hemoglobin; PLT, platelets count; sCD25, soluble CD25.

Table S2. Treatment details and outcome before HSCT.

| Case | Dose of emapalumab | Dose of ruxolitinib (modified#) | Concomitant treatment | Response | Duration of response (weeks)* | Salvage chemotherapy | Outcome |
| --- | --- | --- | --- | --- | --- | --- | --- |
| 1 | 50mg | 15mg bid | - | NR (normal temprarure) | - | DEP regimen | Death |
| 2 | 50mg*2 | 15mg bid | MP (1mg/kg), PD-1 blockade, CRRT | Improvement | 11.3 | DEP regimen | Allo-HSCT |
| 3 | 50mg*2 | 15mg tid | - | PR | 2.8 | Hyper-CVAD A; L-DEP regimen | Death |
| 4 | 50mg | 15mg bid | PD-1 blockade | PR | 10.4 | DEP regimen | HSCT |
| 5 | 50mg | 15mg tid | - | CR | 21.1 | - | Survival |
| 6 | 50mg | 15mg bid | PD-1 blockade | NR | - | VP-16+Chidamide | Death |
| 7 | 100mg | 15mg bid | VP-16 (50mg/m^2^) | Improvement | 1.7 | Hyper-CVAD A | Allo-HSCT |
| 8 | 50mg | 15mg bid | Venetoclax (25mg) | CR | 29.8 | - | Survival |
| 9 | 50mg | 15mg bid | - | PR | 1 | VP-16 | Allo-HSCT |
| 10 | 50mg*2 | 15mg bid | - | CR | 19.9 | - | Survival |
| 11 | 100mg*2 | 10mg bid | PD-1 blockade, CRRT, VP-16 (50mg/m^2^), DXM (2.5mg/m2), IVIG | CR | 18.9 | - | Allo-HSCT |
| 12 | 50mg *2 | 5mg bid | DXM (5mg/m^2^) | NR (normal temprarure) | - | DEP regimen+Rituximab+PD-1 blockade | Survival |
| 13 | 50mg*4 | 15mg bid | VP-16 (50mg/m^2^), MP (0.5mg/kg) | PR | 3.9 | - | Death |

# The dose of ruxolitinib was modified based on the CYP450 inhibitors’ interaction; * The duration of response was calculated from the administration of emapalumab to the loss of response (including relapse of HLH, performance of HSCT or death), or last follow-up.

MP, methylprednisolone; DXM, dexamethasone; IVIG, intravenous immunoglobulin; DEP regimen, doxorubicin-etoposide-methylprednisolone.

Figure S1. Changes in laboratory findings after initiation of ruxolitinib and emapalumab over time. Data for all patients treated on-protocol included (n=13). (A) Temperature (degree Celsius). (B) Absolute platelet count. (C) Absolute neutrophil count. (D) Ferritin concentration. (E) Soluble IL-2 receptor concentration. (F) Alanine transaminase. The green lines and dots represent the data for response-patients, while the red lines and dots represents the data for non-response-patients.


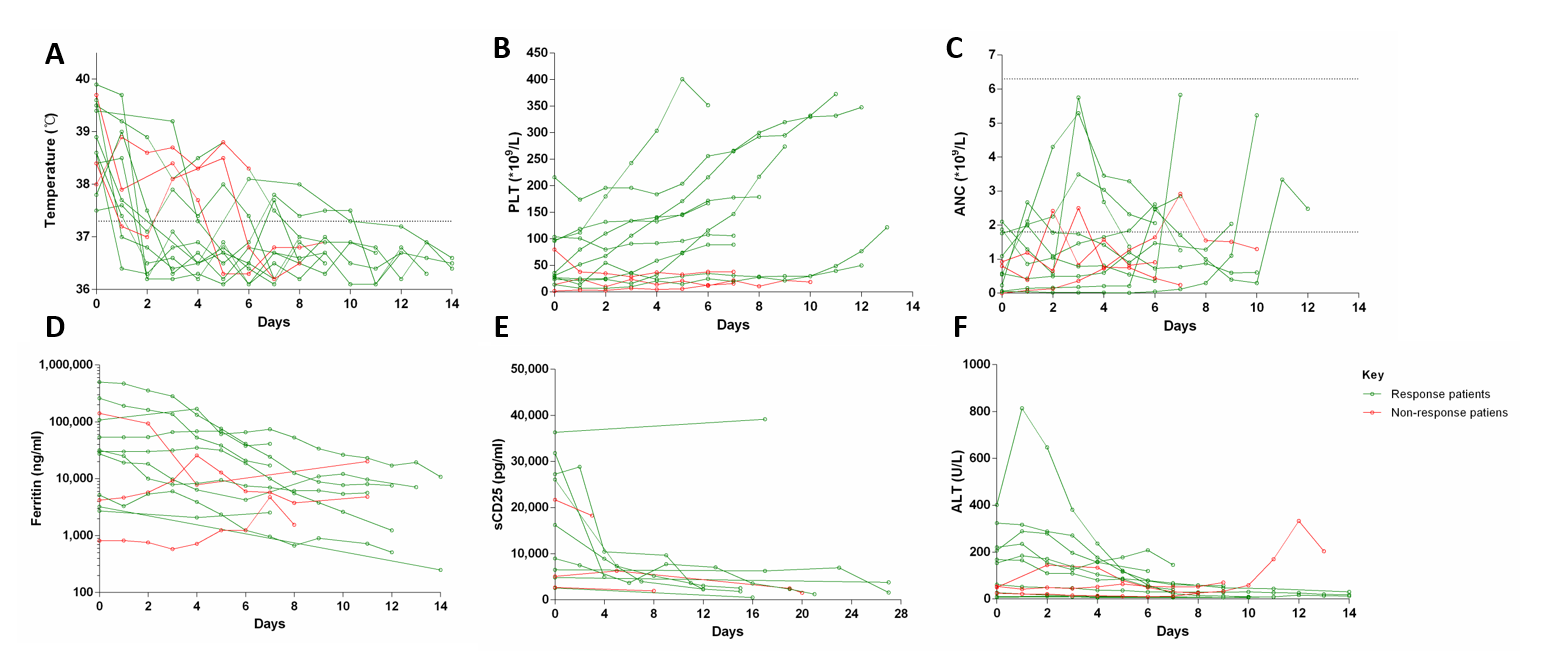


Figure S2. Changes in interferon-γ concentration.Insert shows in detail changes from baseline to day 5 for patients with baseline levels of IFN-γ <2,500 pg/mL.


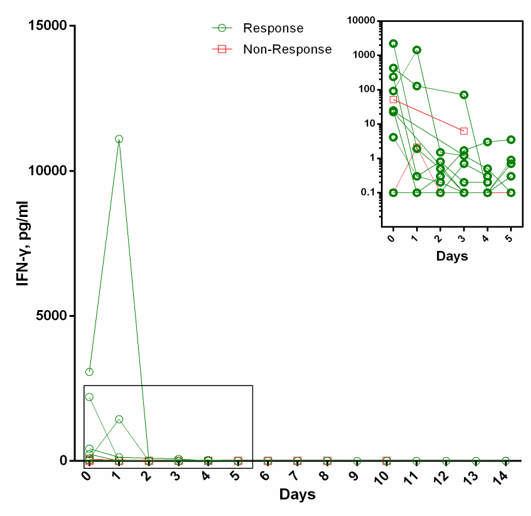


Figure S3. Changes in interleukin-6 concentration. Insert shows in detail changes from baseline to day 5 for patients with baseline levels of IL-6 below 500 pg/mL.


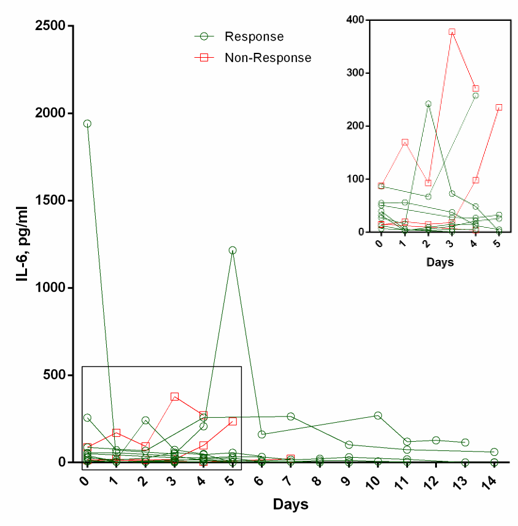


Figure S4. Changes in interleukin-10 concentration. Insert shows changes in IL-10 levels in three additional patients whose data were not shown on the original figure.


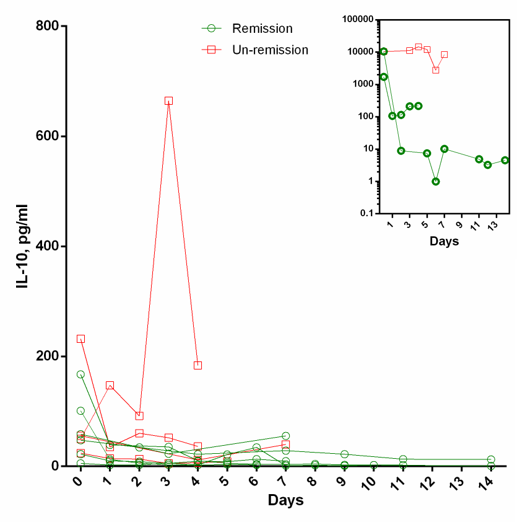

Supplement: Supplementary file 1 — supplementary appendix [file 41408_2024_1056_MOESM1_ESM.docx]
